# An open democracy

**DOI:** 10.1186/1476-4598-6-43

**Published:** 2007-07-03

**Authors:** Shawn Mathur, Christian Schmidt

**Affiliations:** 1 Section of Molecular Genetics and Microbiology and Institute for Cellular and Molecular Biology, University of Texas, 1 University Station, A 5000, Austin, TX 78712, USA; 2 Molecular Cancer, BioMed Central Ltd., Middlesex House, 34-42 Cleveland Street, London W1T 4LB, UK

## Abstract

Sovereign power is retained and shared by the citizens of a country. Using electoral tools, governing structures are formed to ensure protection of national interests. As with any institution, proper control of the government guarantees its adherence to the tasks delegated to it by its citizens. In turn, citizens have to be provided with, and are encouraged to access and evaluate, information generated by the government. On the other hand, governments generate sensitive information (e.g., intelligence, internal reports, etc) that are required for self-evaluation and defense against threats to the nation. Governments are granted a privilege to collect, store and use such information to perform necessary tasks. How far does governmental privilege go relative tothe intrinsic right of citizens to access and evaluate information?

## 

As with all structures and agencies, governmental establishments tend to grow and develop a culture of their own. This is fueled, at least in part, by theendeavor of citizens to be employed by the government and, consequently results in expanded governmental tasks. A growing agency, or governmental structure, does not necessarily require an expansion of the governmental privilege. It is assumed that personal information of governmental employeesis not treated differentlythan that ofemployees of the private sector, implying that all personal information is protected by law.

Given the globalized production and trade of goods and information, governmental structures are poised to become highly centralized and increasingly powerful. Currently, much information is stillarchived using paper files and/or hidden on servers unavailable to mainstream search engines. In addition, citizens feel unable to manage the vast amount of information provided by government agencies. They see a lack of freely available literature as a reference for the formation of an opinion and, ultimately, for offering the government critical input. Withan increased number of citizens sharing sovereign power, an unfettered flow of free information (preferably generated and stored electronically and accessible to every citizen) could be the backboneof the democracy of the futureand hence, the future gold standard of good governance and citizenship. Despite the obvious benefits of electronic information, the efforts of maximizing access to the Internet, which require managing, storing and maintaining information with appropriate security, pose challenges to current technology.

Living in a democratic society comes with a wealth of benefits. Taking the offered benefits and neglecting rights and obligationsthreatens democracy. Failure to exercise the right and duty to evaluate the government and participate in elections results in an electoral representation of a fraction of the citizens of a country. Special interest groups, previously regarded as the minority, are poised to become central to themajority of the active electorate. It is easy to see that campaign strategies that target selected electoral groupsenjoy higherefficiency and may produce a feedback loop, allowing non-mainstream ideas/trends to gain anelevated influence. Once manifest in coalitions and/or governmental policies, this evokes the critique of citizens and the feeling that individual voices do not count. In the extreme, opposing parties may form cartels to secure theirgraspon power. This departure from the original political conviction and the desire to occupy the mandate for a prolonged time can result in an increased number of votes for non-mainstream parties and/or radical alternatives. Inevitably, this shake-up of the establishment results in a more genuine representation of the concerns of citizens in the long run, while imposing inconveniences over the short course. Taking a more philosophical approach, this is simply a wake-up call, issued by theelectorate, and intended to re-vitalize democracy inits true meaning, assuming that democratic forms of self-governance are not obsolete. As stated at the beginning of this paragraph, participation, by any means, is essential for a healthy democracy and simply blaming the government forpoor decisions has little merit as long as the participation of the full electorate in self-governance is missing.

## Conclusion

Why is there so little participation in self-governance? When confronted with a comfortable sofaversus ahard chair in a public hearing and seemingly endless discussions about alternatives, one may simply trust the elected representative to do the right thing. To avoid an obvious argument, elected representatives are fallible as any ordinary citizen and depend on critical evaluation and information from the constituency. Is it a coincidence that electoral districts with little or no communication with representativeshave more frustrated citizens? Is it that the choice of convenience, hence lack of control, leaves elected representatives to the influence of lobbying groups? Finally, how much are we willing to pay for convenience? Arguing furtherthatevery aspect of a givenpolitical platform can rarely be captured in a slogan, simplified messages are often used to substitute for the vast amount of information, available to everyone. Leaving the problem of organizing, storing, presenting and accessing information aside, as well as the dilemma of ad-hoc decisions versus long-term planning, the paradox ofcapturing the interest of the electorateand dealing with a complex matter has to be addressed. A rebuttal for this, although far from comprehensive, is that scholars have the obligation to make their findings accessible to the public, free of charge, in a way such that newspapers and/or other appropriate media arethen able topresent alternatives, new trends and critique of current main-stream endeavors to the broader public, while referring to scholarly papers and/or other articles. As with the Open Access movement gaining momentum, one may see the Internet, in its neutrality, as a forum for discussion and gathering information, or, in other words, the library of the public.

## Competing interests

SM declares that there are no competing interests. CS is deputy editor of *Molecular Cancer *and receives no remuneration for his efforts.

## Authors' contributions

SM and CSdrafted, finalized and approved of the final form of this manuscript. Both authors read and approved the final manuscript.

